# Unbiased discovery of autoreactive type 1 diabetes T-cell receptors that bind specific hybrid insulin peptides

**DOI:** 10.21203/rs.3.rs-10074672/v1

**Published:** 2026-07-14

**Authors:** Rebecca Elyanow, Amanda J. Moore, Tim Hayes, Enrique Crespo, Erica L. Gumucio, Melanie Laur, Sami Rantisi, Ninnia Lescano, Brad Greenfield, Patrick Monnahan, Brian C. Zhang, Rebecca B. Harris, Edward J. Osborne, Ryan Brown, Krystin Samms, Joanna Maltbaek, Alex Dahmani, Todd M. Brusko, Aaron Michels, Mikael Knip, Sharon Benzeno, Bryan Howie, Mark Klinger, Harlan Robins

**Affiliations:** 1 Adaptive Biotechnologies, Seattle, WA, USA.; 2 Department of Pathology, Immunology, and Laboratory Medicine, Diabetes Institute, College of Medicine, University of Florida, Gainesville, FL, USA.; 3 Barbara Davis Center for Diabetes, University of Colorado School of Medicine, Aurora, CO, USA.; 4 Hospital for Children and Adolescents, University of Helsinki, Helsinki, Finland.; 5 Turku Bioscience Centre, University of Turku and Åbo Akademi University, Turku, Finland.

## Abstract

While recent studies have revealed much about the pathogenesis of type 1 diabetes (T1D), the self-antigens and immune receptors that drive cellular autoimmunity have not been precisely determined. In this study, we identify disease-associated T-cell receptor (TCR) sequences, provide evidence that they are involved in T1D pathogenesis, and connect them to specific antigens implicated in beta-cell autoimmunity. We discovered 264 T1D-associated TCRs by comparing TCR repertoires in blood from T1D cases and controls, without any bias toward predefined cell phenotypes or antigens. Multiple lines of evidence support the relevance of these TCRs to T1D pathogenesis: they form convergent sequence clusters linked to the class II HLA risk alleles HLA-DQ8 and HLA-DQ2.5, are found almost exclusively in T1D cases and largely absent in matched controls, can be detected in blood before autoantibodies, have baseline frequencies that stratify post-treatment clinical outcomes, and are enriched in disease-relevant tissues with effector phenotypes; by contrast, the same TCRs, when rarely detected in healthy controls, more often exhibit regulatory phenotypes. We reverse engineered (deorphanized) these TCRs to find their antigenic targets using nucleic acid-based and peptide-based workflows. These independent approaches converged on a narrow set of antigen targets across TCR clusters: a C-peptide-derived hybrid insulin peptide (HIP) hotspot, with representative receptors preferring HIPs over native peptides and remaining specific in proteome-scale testing. Together, these findings connect multiple threads from the T1D literature: the central importance of effector T cells to disease pathogenesis; the role of insulin-derived hybrid peptides as neoantigens; and the potential to use public clusters of TCRs to serve as biomarkers, guide antigen discovery efforts, and elucidate disease mechanisms.

## Introduction

Type 1 diabetes (T1D) is a chronic autoimmune disease in which immune-mediated beta-cell destruction leads to progressive insulin deficiency and eventually, overt hyperglycemia. Convergent genetic, tissue, and functional evidence place T cells at the center of T1D pathogenesis. The strongest inherited risk maps to HLA class II haplotypes, especially DQ2 and DQ8-linked backgrounds, and both blood- and tissue-based studies have documented structured autoreactive T-cell populations rather than a diffuse, nonspecific perturbation. Those observations raise the possibility that public T cell receptor (TCR) sequences might serve simultaneously as clinically useful biomarkers and as clues to the underlying antigenic process^1–12^.

Among candidate antigens, hybrid insulin peptides (HIPs) are especially compelling because they provide a mechanistic bridge between beta-cell biology and recurrent CD4 T-cell recognition. HIPs arise through peptide-fusion events in beta-cell secretory granules and have been recognized as disease-relevant neoepitopes in both mouse and human T1D^13,14^. Subsequent studies have broadened the human HIP repertoire, highlighted the insulin secretory granule as a hotspot of autoantigen formation, shown that proinsulin C-peptide (herein referred to as C-peptide) is a major source of human DQ8-restricted HIPs, and demonstrated HIP-reactive T-cell responses before clinical diabetes^8,11,13–18^. Although the number of theoretically possible HIPs is large (thousands) there is limited information on which HIPs are driving the T cell response in T1D.

Our goal in this study was to more precisely define the T cells (and TCRs) that underlie T1D, and the antigens that activate them. Our approach is based on the idea that public disease-associated TCRs can be captured directly from population-scale blood repertoires, and that these TCRs can yield robust insights about clinical progression and disease mechanism^19–22^. Throughout, we attempt to minimize biases toward existing hypotheses: our case/control approach with rigorous validation finds disease-associated TCRs that generalize beyond the training data, and we present multiple lines of evidence that these TCRs are involved in disease pathogenesis and clinical progression. In addition, we map these TCRs to antigenic targets using two independent workflows (nucleic acid-based and peptide-based deorphanization) that converge on a single answer: CD4 effector T cells encoding these TCRs bind HIPs derived from the proinsulin C-peptide. This result ties together multiple findings from the T1D literature and points the way toward more precise biomarkers and treatments^8,15,23^.

## Results

### A three-stage study design identifies and locks a public TCR biomarker for T1D

**Figure 1 |:**
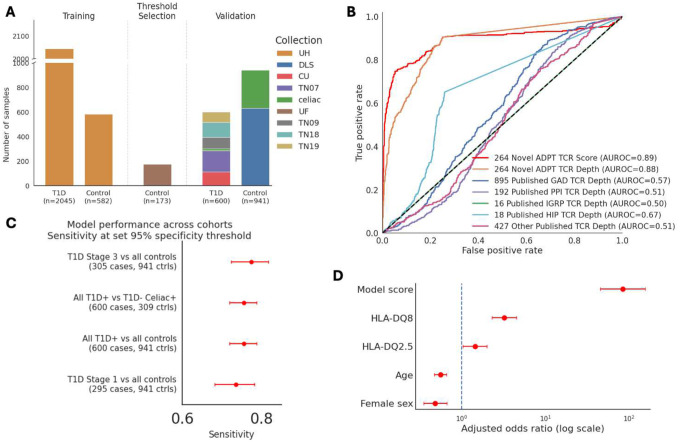
Discovery, threshold locking and independent validation of a public TCR biomarker for type 1 diabetes (T1D). **A,** Study design. Training was performed in the UH cohort (T1D, *n* = 2,045; controls, *n* = 582). A fixed classification threshold was selected using an independent UF control set (*n* = 173). Locked evaluation was performed in external cohorts (T1D, *n* = 600; controls, *n* = 941) comprising DLS, CU, TrialNet study (TN07, TN09, TN18, TN19) and celiac disease controls. **B,** Receiver operating characteristic (ROC) curves comparing the 264-receptor Adaptive TCR score and corresponding clonal-depth model with previously published T1D-associated TCR sets. **C,** Sensitivity at a fixed threshold corresponding to 95% specificity (defined in the UF control set), evaluated in prespecified comparisons: stage 3 T1D versus all controls (305 cases, 941 controls; sensitivity 0.77, 95% CI 0.72–0.82); all T1D versus celiac controls (600 cases, 309 controls; 0.76, 0.72–0.79); all T1D versus all controls (600 cases, 941 controls; 0.76, 0.72–0.79); and stage 1 T1D versus all controls (295 cases, 941 controls; 0.74, 0.68–0.78). **D,** Multivariable logistic regression showing adjusted odds ratios for T1D, including the repertoire score, HLA-DQ8, inferred HLA-DQ2.5, age and sex (log scale).

The analysis implemented a three-stage framework intended to prevent information leakage into the evaluation. After sample-level quality control requiring productive rearrangements > 50,000, the training set comprised 2,627 samples from the University of Helsinki (UH), including 2,045 stage 3 T1D cases within 100 days from diagnosis and 582 controls (Figure 1A). The cases and controls in the training set were well matched in age, sex, and DQ8 status (p>0.01), with a slight skew towards DQ2.5 and DRB1*04:01 (DR4) in the cases (p<0.01, Figure S1). The threshold-selection set comprised 173 T1D-negative controls from University of Florida (UF). The holdout set comprised 600 stage 3 (within 100 days from diagnosis) or stage 1 T1D samples collected from Colorado University (CU) and baseline samples from TrialNet studies TN07, TN09, TN18, TN19, 632 healthy controls from Discovery Life Sciences (DLS), and 325 Celiac-positive controls from City of Hope (600 T1D cases and 941 controls overall) (Table S1). For all samples, PBMCs were collected and sequenced on the immunoSEQ Assay (see Methods). Public T1D-associated TCRs were identified as those with significantly higher presence in cases vs controls in the UH training set, with parameters selected to optimize sensitivity at 95% specificity in 5-fold cross-validation (see Methods). This yielded 264 public T1D-associated TCRs that we will from now on denote the ADPT TCR set (Table S2).

In the locked evaluation, the model score, defined using only two features (frequency of ADPT TCRs and total productive templates), achieved AUROC 0.89 in holdout testing (Fig. 1B). Using the threshold selection cohort comprising 173 controls, all AAb- relatives of someone with T1D, we locked a model score threshold of 2.13 at 95% specificity (Fig. S3). At this threshold, sensitivity in the untouched holdout cohorts was 75.5% for all T1D cases versus all controls, 73.6% for stage 1 disease versus all controls, 77.4% for stage 3 disease versus all controls, and 75.5% for all T1D cases versus celiac-positive controls (Fig. 1C).

To assess whether this strong signal was confounded by T1D-associated covariates (HLA risk alleles, age, and sex), we performed two complementary analyses. First, we fit a logistic model including standardized model score, HLA-DQ8, HLA-DQ2.5, age, and sex. After adjustment, model score remained the dominant predictor of case status (OR 84.7), exceeding the contributions of DQ8 (OR 3.23) and DQ2.5 (OR 1.44).

Second, we evaluated classifier performance within covariate-defined subgroups. Model performance remained strong across HLA-defined strata, including DQ8+, DQ8−, DQ2.5+, and DQ2.5− individuals (AUROC range 0.80–0.92), as well as across age and sex strata (Fig. S2). Notably, the classifier retained discriminative ability in individuals lacking both DQ8 and DQ2.5 (AUROC = 0.76; 62 cases, 417 controls), indicating that the repertoire signal is not solely driven by these major HLA risk haplotypes. Together, these analyses demonstrate that the locked repertoire score captures disease-relevant information not reducible to age, sex, or major HLA enrichment alone.

To assume an exact comparison to published TCR sets, we computed clonal depth (see Methods) for our ADPT TCR set and compared it to published TCRs binding known T1D-associated antigens, including GAD-, insulin/PPI-, IGRP-, HIP-, and other literature-derived receptor sets^23,24^ (Table S3). TCRs that are listed as binding multiple antigens are included in both sets. Our ADPT TCRs achieved AUROC 0.88, whereas the published comparators ranged from 0.50 to 0.67. Notably, the most predictive published TCR set was the 18 HIP-associated TCRs. This was also the only published set to intersect with our 264 ADPT TCRs, with TCR “CASSLGPGARETQYF+TCRBV05–01+TCRBJ02–05” present in both sets. Together, these results demonstrate that the ADPT TCR set provides substantially improved discriminative performance over existing antigen-specific TCR collections, underscoring its potential utility for more accurate identification of T1D-associated immune signatures.

### T1D-associated TCRs form public convergent clusters structured by HLA

**Figure 2 |:**
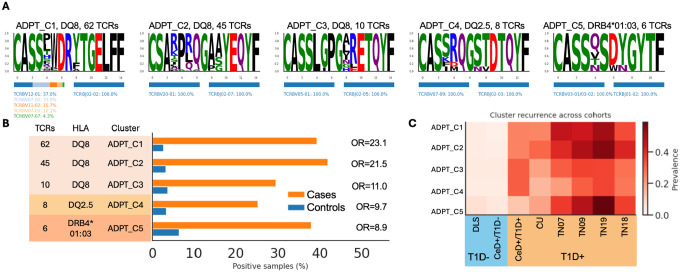
Disease-associated public TCRs form convergent HLA-associated sequence clusters. **A,** Sequence logos for the five public TCR clusters most strongly associated with T1D (ADPT_C1–ADPT_C5). Cluster size, case–control odds ratio (OR), false discovery rate-adjusted *q-*value and dominant inferred HLA association are shown above each logo. TRBV and TRBJ gene usage frequencies are shown below. **B,** Prevalence of the five clusters in T1D cases and controls in the independent holdout cohort. Numbers at right indicate case–control odds ratios for each cluster. **C,** Recurrence of the five clusters across independent cohorts. Heat map indicates cluster prevalence within each cohort.

The ADPT TCR set resolves into convergent CDR3 motif clusters that are strongly enriched in cases and show clear HLA restriction. Five dominant clusters account for a large fraction of this signal (Fig. 2A,B): ADPT_C1 (n = 62, OR = 23.8; DQ8), ADPT_C2 (n = 45, OR = 17.8; DQ8), ADPT_C3 (n = 10, OR = 5.8; DQ8), ADPT_C4 (n = 8, OR = 5.6; DQ2.5), and ADPT_C5 (n = 6, OR = 4.2; DRB4*01:03).

These clusters were consistently detected across independent T1D cohorts while remaining sparse in controls (Fig. 2C), indicating that the associated receptors represent shared (“public”) immune responses rather than private or cohort-specific expansions. Beyond these dominant clusters, additional receptors in the ADPT set exhibit similar motif convergence and HLA association (Fig. S4), suggesting that this structure extends beyond a small number of high-prevalence clones.

Collectively, these features argue against a diffuse bystander perturbation of the repertoire and instead support a focused, antigen-driven response characterized by convergent recombination and HLA restriction. The degree of public convergence is notable given prior difficulty in reproducibly detecting blood-based autoreactive T cell signals in T1D^6,23,25,26^.

### T1D-associated TCRs are detected early in the DAISY cohort

We sequenced prospectively sampled PBMCs from The Diabetes Autoimmunity Study in the Young (DAISY), including 27 subjects who were later diagnosed with T1D and 25 controls^7,25,27,28^. Individuals who progress to T1D show elevated levels of our novel T1D-associated TCRs, while those who do not progress have nearly undetectable levels, with an average model score (Z score) of 2.96 for eventual T1D converters (above the 95% specificity threshold) and −0.01 for non-converters. The full timeline from birth to T1D diagnosis or final timepoint, including the timing of seroconversion (presence of 1 or more positive autoantibody titers) and T-cell positivity (T1D model score above the 95% specificity threshold), indicates that in many subjects T-cell positivity is observed prior to seroconversion (Fig. S5). Notably, T-cell score was assessed at only four visits per subject, whereas antibodies were measured approximately annually (median of 12 follow-up visits per subject), suggesting that observed lead times are conservative given denser sampling of serologic measurements.

**Figure 3 |:**
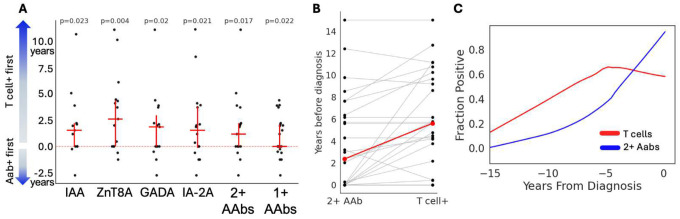
T1D-associated TCRs are detected before seroconversion in the DAISY cohort. **A,** Differences in years-before-diagnosis between first TCR positivity and first positivity for insulin autoantibodies IAA, ZnT8A, GADA, IA-2A, ≥2 autoantibodies or ≥1 autoantibody. Positive values indicate earlier detection of the TCR signal relative to autoantibody positivity. Points represent individual participants; red markers and error bars indicate medians and interquartile ranges. *P* values are shown above each comparison. **B,** Paired within-participant comparison of years-before-diagnosis for first detection of ≥2 autoantibodies and first TCR positivity. Red markers indicate cohort medians. **C,** LOWESS-smoothed prevalence curves showing the fraction of participants positive for the TCR signal or ≥2 autoantibodies over time before diagnosis. The TCR signal increased earlier than the ≥2 autoantibody state. TCR positivity was defined using the locked threshold established in the UF control cohort.

We next quantified lead time, defined as the difference in years between first T-cell positivity and first detection of each serologic milestone within subjects. T-cell positivity was detected earlier than insulin autoantibody (IAA), zinc transporter 8 autoantibody (ZnT8A), glutamic acid decarboxylase autoantibody (GADA), insuloma-associated protein 2 autoantibody (IA-2A), the two-autoantibody state that defines stage 1 disease, and the first-autoantibody state (Figure 3A). Median lead times were positive for all six serologic milestones, with the largest lead time observed for ZnT8A (p=0.004, Wilcoxon signed-rank test). Paired within-subject analyses favored earlier T-cell detection in each comparison (Fig. 3B, Fig. S6). A LOWESS-smoothed curve shows that the fraction of T-cell-positive samples rises earlier than the two-autoantibody state and peaks prior to stage 3 T1D diagnosis, whereas serologic positivity continues to accumulate closer to diagnosis (Fig. 3C).

To examine timing relative to disease onset, we plotted model score against years from stage 3 diagnosis in prediagnostic and early postdiagnostic samples (within 100 days from diagnosis). In prediagnostic samples, the TCR model score increased as samples approached diagnosis (Spearman rho = 0.25, p = 0.001). In postdiagnostic samples collected within 100 days of diagnosis, the model score decreased with increasing time from diagnosis (Spearman rho = 0.16, p = 0.028) (Fig. S7). Together, these findings indicate that the T-cell signature rises in the years preceding diagnosis, peaks near onset, and contracts shortly thereafter.

### Baseline T-cell score is retrospectively associated with outcome in independent Abatacept studies

The TrialNet studies TN09 and TN18 evaluated abatacept in stage 3 and stage 1 disease, respectively^3,29^. In TN09, the subject-level endpoint was 24-month C-peptide area under the curve (AUC), modeled as log(C-peptide AUC at 24 months + 1) with terms for treatment, baseline T cell subgroup, their interaction, baseline C-peptide AUC, and age. In TN18, time to progression to stage 2 or stage 3 T1D was analyzed using a 6-month person-period complementary log-log model including treatment, baseline T cell subgroup, age, and interval indicators.

We assessed whether baseline T cell score identified subgroups with differential outcome trajectories. In TN09, the model-adjusted abatacept-to-placebo (A/P) ratio was higher in the low-baseline-score subgroup than in the high-score subgroup (A/P = 4.46 vs 1.02; threshold-interaction p = 0.049; Fig. S8A). In TN18, event-free survival curves showed greater separation between treatment arms in the low-baseline-score subgroup, although the interaction did not reach statistical significance (RMST interaction p = 0.057; Fig. S8B).

Consistent with these patterns, formal interaction testing showed a positive treatment-by-subgroup effect in TN09 (β = 0.413, 95% CI 0.046–0.781; two-sided p = 0.027; n = 61) and a directional effect in TN18 (95% CI −3.3 to 45.2; permutation p = 0.076; n = 83), both favoring greater benefit in the low-baseline-score subgroup. Cross-trial aggregation yielded a weighted Stouffer Z of 2.78 (two-sided p < 0.005; Fig. S8C). These data suggest that measuring the presence of our novel T1D TCRs in the blood prior to treatment may capture clinically relevant heterogeneity in treatment response among at-risk and newly diagnosed individuals.

### T1D-associated TCRs are in T cells with a conventional CD4 phenotype from relevant tissues

**Figure 4 |:**
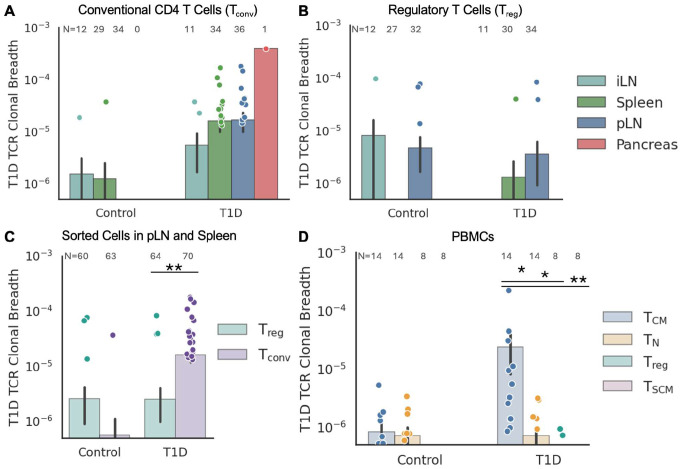
T1D-associated TCR breadth is enriched in conventional and memory CD4 T cell compartments across lymphoid tissues and blood. **A,B,** TCR clonal breadth matching the T1D-associated repertoire in sorted conventional CD4 T cells (T_conv_) (**A**) and regulatory T cells (T_reg_) (**B**) from pancreatic lymph node (pLN), spleen, inguinal lymph node (iLN) and pancreas in donors with T1D and controls. Numbers above each group indicate samples with detectable T1D-associated clonotypes (at least one clone) over total samples analyzed. **C,** TCR clonal breadth in sorted T_reg_ and T_conv_ populations isolated from pLN and spleen of donors with T1D and controls. **D,** TCR clonal breadth in peripheral blood mononuclear cell (PBMC) subsets, including central memory (T_CM_), naive (T_N_), regulatory (T_reg_) and stem cell memory (T_SCM_) T cells, from donors with T1D and controls. Points represent individual samples; bars and error bars indicate means ± s.e.m. Statistical comparisons were performed using two-sided Mann–Whitney U tests; *P* < 0.01 (*), *P* < 0.001 (**).

To determine whether our novel T1D-associated TCRs are preferentially localized to disease-relevant compartments, we quantified clonal breadth across sorted CD4 T cell subsets from pancreas, pancreatic lymph node (pLN), spleen, inguinal lymph node (iLN), and peripheral blood (PBMCs)^30,31^. The TCRs were enriched in conventional CD4 T cells (T_conv_) from pancreas, pLN, and spleen of donors with T1D, whereas these TCRs were largely absent from the tissues of matched controls (Fig. 4A). In contrast, regulatory T cells (T_reg_) showed little enrichment in either group (Fig. 4B).

Consistent with this, the relative contribution of T_conv_ versus T_reg_ compartments was significantly shifted toward T_conv_ in spleen and pLN from T1D donors, while controls showed comparatively higher representation within the T_reg_ compartment (Fig. 4C). In PBMCs, our novel T1D-associated TCRs were concentrated in the central memory (T_CM_) CD4 compartment in T1D donors, with sparse detection in naïve cells and no enrichment in T_reg_ or stem cell memory (T_SCM_) subsets (Fig. 4D). Matched controls showed little enrichment for these TCRs in any compartment. Together, these data indicate that T1D-associated TCRs are enriched in anatomically relevant lymphoid tissues and preferentially reside in conventional and memory CD4 T cell compartments, rather than regulatory T cells.

### Deorphanization reveals clustered HIP antigen targets of T1D-associated TCRs

Our TCR deorphanization workflows utilize either nucleic acid-based libraries or combinatorial peptide libraries to identify the antigenic targets of ADPT TCRs. In the first approach, we generated a nucleic acid library comprising 3,211 oligonucleotides encoding HIPs and beta-cell–derived proteins (plus other published antigens implicated in T1D) and a human proteome library consisting of 633,335 oligonucleotides spanning the human proteome. Antigen-presenting reporter cells (APrCs) were pre-engineered to express an IL-2 capture receptor and a defined HLA class II molecule (HLA-DQ8, HLA-DQ2.5, or HLA-DRB4), with separate library transductions performed for each HLA.

Effector CD4+ Jurkat T cells expressing ADPT TCRs were co-cultured with library-transduced APrCs for 16 hours. Antigen-specific activation was determined by IL-2 secretion and surface capture on the subset of APrCs presenting antigens that elicit a response from the T cells. IL-2 labelled APrCs were enriched by magnetic selection and fluorescence-activated cell sorting and sequenced to quantify enrichment of encoded antigens.

**Figure 5 |:**
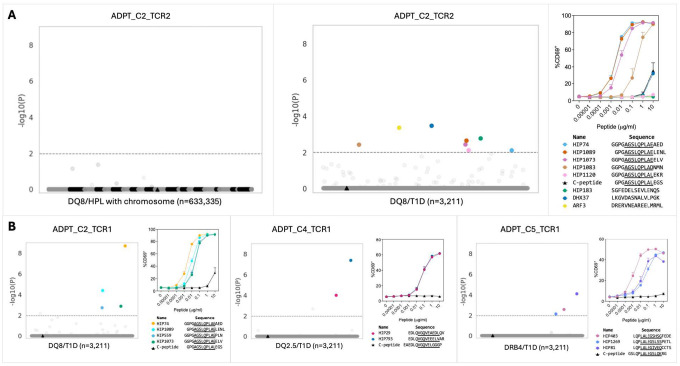
Deorphanization workflows reveal novel targets of T1D-associated TCRs **A,** A DQ8 restricted T1D TCR tested against nucleic acid libraries encoding 50 amino acid segments either tiled across the entire human proteome (left) or a defined set of antigens implicated in T1D including HIPs (center) enriched antigens derived from the T1D library but not the human proteome. Enrichment p-values (Family-wise error rate adjusted) for each tested library segment are reported in corresponding genome order with library-wide significance marked by the dashed horizontal line. For reference, C-peptide (not enriched) is noted with a black triangle in the enrichment plots at left and center. Highlighted segments (colored points) were validated with peptide titrations (right) of the putative 15AA sequence derived from the enriched segments at varying concentrations (x-axis) using CD69 upregulation of transfected Jurkat T cells harboring the TCR of interest. Response to C-peptide, not enriched from either library, was included as a reference. The predicted core binding sequence of each epitope is underlined. **B,** T1D TCRs restricted by three different HLAs (DQ8, DQ2.5 and DRB4, respectively) were used to query APrCs expressing a T1D antigen library consisting largely of HIPs and beta-cell-derived proteins. In all three queries, the most significantly enriched sequences (colored points) were those that contained HIPs with each converging on a distinct cluster-specific target motif. Titrations of these HIPs (inset) were validated as in (A) along with their corresponding portion of C-peptide revealed strong TCR reactivity to the HIPs but not C-peptide.

In a representative experiment, T1D and human proteome library-expressing APrCs were queried with an HLA-DQ8-restricted ADPT TCR, ADPT_C2_TCR2 (cluster ADPT_C2; Fig. 5A, left and middle). Significant enrichment (Family-wise error rate adjusted p-value < 0.01) was observed exclusively among APrCs transduced with the T1D library. Of the eight enriched sequences, six contained HIPs and two corresponded to germline-encoded peptides. Notably, these two germline sequences were also present in the full human proteome library but were not enriched. Five of the eight enriched sequences shared an 8-mer motif (AGSLQPLA), corresponding to the top predicted 9-mer core (AGSLQPLAE) identified using a parallel positional scanning synthetic combinatorial library (PS-SCL) methodology^32^ (Supplemental Fig. 13). Remarkably, no additional significantly enriched sequences were detected when T cells harboring this TCR were co-cultured with APrCs transduced with the full human proteome library containing 633,335 sequences. The top eight enriched sequences were ordered as peptides and used in T cell activation assays to confirm the specific reactivity of ADPT_C2_TCR2 for AGSLQPLA{ED}-containing peptides (Fig. 5A, right).

Screening the HLA-DQ8-expressing T1D library with another ADPT TCR from cluster ADPT_C2, ADPT_C2_TCR1, resulted in the significant enrichment of four HIPs also containing the AGSLQPLAE motif (Fig. 5B, left). Given the similarity in TCRα and TCRβ chains between ADPT_C2_TCR2 and ADPT_C2_TCR1 (Table S4), this convergence on a shared epitope was expected.

ADPT TCRs restricted by HLA-DQ2.5 and HLA-DRB4*01:03 (DRB4) similarly showed enrichment for sequences containing HIPs, with distinct motif preferences: VGQVExExx and LALEGxxxx, respectively (Fig. 5B, middle and right). Notably, the most enriched sequences for each TCR were predominantly HIP-derived. TCRs were more functionally avid to the HIPs derived from these enriched sequences, each centered around a common 9mer core epitope, and were either non- or weakly-responsive to homologous germline-encoded C-peptide (Fig. 5).

Control experiments were performed to confirm functional dependence on expression of the correct HLA on APrCs and correct TCRαβ pairing. No antigens were enriched when an HLA-DQ8-restricted ADPT T1D TCR was used to query APrCs expressing HLA-DRB4. Additionally, when the TCRβ of an ADPT T1D TCR was paired with an alternate TCRα, no antigens were enriched (Supplemental Fig. 11).

To corroborate the results above we also performed peptide-based TCR deorphanization using PS-SCL on a subset of ADPT TCRs. The peptide library was designed for HLA-DQ8-restricted TCRs using a fixed base peptide design, GG{EA}xx{GLF}xPxxEGG, with variable positions sampled across all amino acids. A T-cell activation assay was performed with PS-SCL peptides, producing heatmaps depicting CD69 upregulation (Supplemental Fig. 12A, 13A). Despite distinct TCRα and TCRβ chains, TCRs from ADPT_C1 and ADPT_C3 (Fig. 2) converged on similar specificity profiles (Supplemental Fig. 12A). A HIP epitope database was constructed bioinformatically to create spliced peptides of proinsulin to itself or to other beta-cell granule proteins^8,13^. Candidate peptides were filtered by predicted HLA-DQ8 binding (NetMHCIIpan4.3, %EL_rank < 10) and TCR reactivity profiles were used to rank probable epitopes. The top five predicted HIPs for ADPT_C1 and ADPT_C3 shared a common ELGGGPxxE motif (Supplemental Fig 12B). ADPT_C3 TCR, A5.5, responded to all top five predicted HIPs, whereas ADPT_C1 TCRs were more specific for ELGGGPGVE-containing HIPs (Supplemental Fig 12C). T1D TCRs from ADPT_C2 and ADPT_C7 converged on a shared epitope motif, with AGSLQPLAE emerging as the dominant 9mer (Supplemental Fig. 13), consistent with nucleic acid-based TCR deorphanization results (Fig. 5).

Together, these data demonstrate that nucleic acid–encoded library deorphanization of T1D-associated TCRs enables identification of distinct presented HIP-derived antigens across multiple HLA class II contexts. PS-SCL results corroborated these findings for ADPT_C2_TCR1 and ADPT_C2_TCR2 and enabled deorphanization of additional HLA-DQ8-associated TCRs from other clusters (ADPT_C1, ADPT_C3, ADPT_C7). These complementary approaches enable identification of processed and presented epitopes and well-defined TCR-antigen recognition motifs applicable to broader antigen discovery.

### Independent public TCR clusters converge on C-peptide-derived HIP hotspots

**Figure 6 |:**
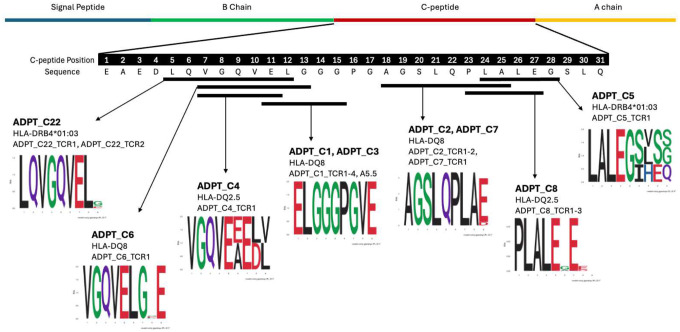
Schematic overview of HIP hotspots within C-peptide. HIP sequences derived from C-peptide were grouped by sequence similarity to highlight shared antigenic motifs recognized by diverse TCRs. For each antigen specificity, the associated TCR cluster(s) and HLA restrictions are indicated. Seq2Logo plots were generated from validated HIPs that induced TCR activation. The map denotes the location within C-peptide from which the N-terminal region of each HIP originates.

Deorphanization enabled identification of distinct HIPs that elicit a response from representative TCRs from all ADPT TCR clusters tested (Fig. 6). HLA-DRB4*01:03-restricted ADPT_C22 TCRs recognized epitopes containing LQVGQVELx (Supplemental Fig. 15), distinct from ADPT_C5_TCR1 recognizing LALEGxxxx motifs (Fig. 5b). Two unique epitope specificities were also identified for HLA-DQ2.5-restricted TCRs: VGQVExExx for ADPT_C4_TCR1 (Fig. 5b) and PLALExExx for ADPT_C8 TCRs (Supplemental Fig. 15).

Among HLA-DQ8–restricted T1D TCRs, three epitope clusters were identified. ADPT_C6_TCR1 recognized VGQVELGxE motifs (Supplemental Fig. 15), arising from similar regions as DRB4- and DQ2.5-restricted epitopes but with distinct junctions and anchor residues. TCRs from different clusters converged on two dominant HIP motifs: ELGGGPGVE (ADPT_C1 and ADPT_C3; Supplemental Fig. 12) and AGSLQPLAE (ADPT_C2, ADPT_C7); Fig. 5a–b, Supplemental Fig. 13).

Both deorphanization approaches consistently demonstrated specificity for HIPs over C-peptide. Germline C-peptide sequences were not enriched in nucleic acid–based library experiments with TCRs derived from clusters ADPT_C2, ADPT_C4 or ADPT_C5 (Fig. 5), and were not predicted as probable peptides by PS-SCL for clusters ADPT_C1, ADPT_C2 and ADPT_C7 (Supplemental Fig. 12–13). Functional assays confirmed negligible or weak responses to C-peptide relative to HIPs. We also evaluated the responsiveness of all TCRs to recombinant proinsulin protein using monoallelic HLA-expressing K562 cells matched to each TCR’s HLA restriction (Supplemental Fig. 14D). B3.1 was used as a control TCR due to its ability to respond to native proinsulin^33^. The majority of the ADPT TCRs did not respond to epitopes derived from K562 cells incubated with recombinant proinsulin protein, supporting their specificity for HIPs. When a response to recombinant proinsulin was observed, it supported the weak C-peptide reactivity detected in T cell activation assays.

By employing a TCR-directed rather than epitope-guided strategy, our deorphanization framework enables unbiased identification of antigenic targets across diverse TCRs. Concordant results across orthogonal assays, coupled with functional validation, define a set of shared HIP epitopes and establish a framework for mapping TCR specificity in T1D.

## Discussion

This study identifies a public TCR signature of T1D that is reproducible across independent cohorts, linked to disease-associated HLA backgrounds, enriched in disease-relevant tissues, and directed against a restricted set of C-peptide-derived HIPs. Using population-scale blood repertoires without predefined antigen selection, we identified shared disease-associated TCRs directly from case-control comparisons and mapped their antigenic targets using orthogonal deorphanization workflows. Both approaches independently resolved to the same antigenic space derived from C-peptide.

The study design is central to this conclusion. Disease-associated TCRs were discovered in one cohort, the classification threshold was locked in an independent control cohort, and all remaining cohorts were reserved for untouched evaluation. The resulting repertoire signal generalized across disease stages, institutions, and control populations, remained associated with disease after adjustment for age, sex, and major HLA risk alleles, and substantially outperformed previously published T1D-associated TCR collections^12,34^.

The convergence on a restricted C-peptide HIP hotspot provides a clear mechanistic context for these observations. HIPs have emerged as important neoepitopes in human T1D and recent work has further implicated the insulin secretory granule as a major site of autoantigen formation, and C-peptide as an important source of DQ8-restricted targets recognized by human islet-infiltrating T cells^8,11,14–18^. The present findings extend that framework by showing public disease-associated TCRs identified from blood independently resolve to the same antigenic space. This is a significant advance in the study of autoimmunity and T1D: it shows that public TCR clusters identified without prior hypotheses about antigen specificity can converge on the same antigenic space, lending further support to a prevailing hypothesis while clarifying details of the molecular interactions that cause the adaptive immune system to break tolerance.

Utilizing our orthogonal deorphanization workflows to query all theoretically possible HIPs, we discovered eight different antigen hotspots. Remarkably, we discovered a pattern of HIPs all stemming from a left fragment of C-peptide, which has been observed in other studies examining HLA-DQ8-restricted CD4^+^ T cells^8,16^. In addition to HLA-DQ8-restricted T cells, we also observe the same trend for HLA-DQ2.5- and HLA-DRB4*01:03-restricted T1D T cells, suggesting a mechanism of HIP generation in this localized area of C-peptide.

Definitive evidence of naturally-occurring HIPs can be provided by mass spectrometry, which is arduous given the amount of tissue material required as well as the ability to confidently identify originating N’ and C’ terminal fragments when the hybrid junction is not centered in the epitope. The ADPT_C2 and ADPT_C7 cluster HIP hotspot, AGSLQPLAE, was found in mass spectrometry results in prior studies^13^, but was disregarded due to the short C’ terminal fragment. The cluster ADPT_C1, ADPT_C3 pattern of T cell specificity, ELGGGPGVE, has been previously observed from human islet-infiltrating CD4^+^ T cells^8,16^. The deorphanization workflow enabled the definition of TCR specificity by both (a) identifying HLA-presented ligands using a nucleic acid-based approach and (b) mapping the potential spectrum of TCR reactivity through the PS-SCL method. Each method independently identified the same target antigens, bolstering confidence in deorphanizing each TCR cluster. Similarly, both approaches allowed us to determine the stringency of TCR specificity for the C-peptide derived N’ terminal portion, as evidenced by a strict left fragment motif. The flexibility in the contributing C’ terminal regions is likely to contribute to neoantigen formation by generating preferred anchor residues for the corresponding HLAs. HLA-DQ8 and HLA-DQ2.5 binding preferences are skewed toward negatively charged amino acids, Glu and Asp. HLA-DRB4*01:03, which is in linkage disequilibrium with HLA-DQ8 and, thus, has a higher likelihood of presence in T1D patients, also exhibits a preference for Glu/Asp in anchor position 7. Germline-encoded C-peptide contains a higher proportion of Asp/Glu than other human proteins, but the spacing does not easily confer HLA presentation. For example, native C-peptide AGSLQPLALE has a much lower likelihood of HLA-DQ8 presentation than the ADPT_C2 and ADPT_C7 HIP hotspot, AGSLQPLAE. Notably, the vast majority of ADPT TCRs do not recognize native C-peptide and, in the rare cases when they do, the response is diminished compared to HIPs.

The longitudinal and clinical analyses further suggest that these public TCRs track biologically meaningful stages of disease progression. In DAISY, the repertoire signal frequently became detectable before serologic milestones within the sampling framework of the cohort and increased approaching diagnosis before contracting after disease onset. In independent TrialNet abatacept studies, baseline repertoire scores retrospectively stratified clinical trajectories. While these findings require prospective validation, they support the possibility that public autoreactive TCRs reflect dynamic pathogenic activity^4,23^.

We acknowledge several limitations to our study. The 264 public receptors identified here are unlikely to represent the full disease-relevant T cell response, particularly across diverse HLA backgrounds. The timing analyses are constrained by sampling cadence. In addition, convergence on a C-peptide HIP hotspot does not exclude the existence of other important antigenic targets in T1D that were not tested. Further, while flow-sorting indicated these cells occupy the CD4 central memory compartment, future studies need to be performed to define the specific cell types.

An important open question is whether the TCRs identified here mark a stable pathogenic population or cells that shift across functional states during progression and after immune intervention. Resolving that will require longitudinal mapping of these receptors’ cell types. Such studies could clarify how the HIP-associated public response is organized across disease stages and whether treatment is accompanied by meaningful shifts in the phenotype of the T cells harboring ADPT TCRs.

Taken together, these findings establish that a public T-cell response to a restricted HIP-associated antigenic space is a reproducible feature of human T1D and can be read out directly from blood. The significance of that result is twofold. First, it shows that a blood-based TCR biomarker can capture disease-relevant biology across independent cohorts, disease stages, and clinical contexts. Second, it shows that an unbiased, hypothesis-free TCR discovery strategy can recover not only a clinically informative receptor signature, but also the shared antigenic specificity underlying that signature. More broadly, this work defines a direct link between population-scale repertoire architecture and disease mechanism, and suggests that public autoreactive TCRs can serve simultaneously as biomarkers, mechanistic probes, and anchors for antigen-specific treatments in T1D.

## Methods

### Data Availability

All data supporting the findings of this study are provided within the article and its Supplementary Information. Source data underlying all main and supplementary figures and tables, including the complete disease-associated TCR set, antigen annotations, cluster assignments, and summary statistics are provided.

### Study Participants and Sample Collection

Written informed consent was obtained from all participants or their legal guardians if the participant was under 18 years of age. Sample collection for the UF cohort was performed as described previously. Sample collection for the CU cohort was performed with study approvals provided by the Colorado Multiple IRB. The TN07, TN09, TN18, and TN19 participants were part of registered clinical trials organized through the NIH TrialNet consortium^2,3,29,35^. All protocol and consent documents were approved by appropriate independent ethics committees or institutional review boards.

### Experimental model and subject details

Overall study design and cohorts. The analysis used a three-stage design consisting of (i) discovery/training in UH samples, (ii) threshold locking in an independent UF control-only cohort, and (iii) locked evaluation in the remaining holdout cohorts. After filtering to productive rearrangements > 50,000, the training set contained 2,045 stage 3 T1D cases and 582 controls from UH, the threshold-selection cohort contained 173 UF controls, and the holdout set contained CU (n = 111), DLS (n = 632), TN07 (n = 174), TN09 (n = 93), TN18 (n = 121), TN19 (n = 85), and celiac (n = 325) samples. Detailed subject characteristics are provided in table S1. In the discovery, threshold-selection, and holdout biomarker analyses, only one sample per subject was used. For TrialNet cohorts, the baseline sample was used for biomarker scoring to avoid confounding by therapeutic exposure. Stage 3 donors were sampled within 100 days of T1D diagnosis.

### TCRB Repertoire Sequencing

Genomic DNA was extracted from frozen, plasma-depleted blood samples using the Qiagen DNeasy Blood Extraction Kit (Qiagen). As much as 18 μg of input DNA was then used to perform immunosequencing of the third complementarity determining (CDR3) region of TCRβ chains using the immunoSEQ Assay (Adaptive Biotechnologies). Briefly, input DNA was amplified in a bias-controlled multiplex PCR, followed by high-throughput sequencing. Sequences were collapsed and filtered to identify and quantify the absolute abundance (i.e. templates) of each unique TCRβ CDR3 for further analysis, as previously described^19–21^. To quantify the proportion of T cells out of total nucleated cells input for sequencing, or T-cell fraction, a panel of reference genes present in all nucleated cells was amplified simultaneously.

### Calculation of repertoire statistics: clonal breadth, clonal depth, and clonality

We define the quantities clonal breadth and clonal depth as described previously^21^, which estimate the relative frequency of unique productive TCRs and clonally expanded productive TCRs respectively. Productive Simpson clonality, a measure of the evenness of clonotype population size, was calculated as previously described^20^.

### Enhanced Sequence Logistic Growth Model (ESLG)

The observed background rate of public disease-associated TCRs is a function of the number of sequenced T cells (total productive templates). Empirically, the background number of ADPT TCRs in our set of training controls fit a logistic-growth curve (Fig. S9). To leverage the number disease-associated TCRs and total productive templates as a diagnostic classifier, we modeled the number of disease-associated TCRs as a logistic-growth function of the number of total productive templates sampled from a repertoire and fit this model to the 582 control repertoires in the training data. The resulting model provides a normalized estimate of the degree to which the disease signature of a case sample deviates from the control data, expressed in standard deviations from the mean.

This approach carefully controls specificity by considering hundreds of control repertoires. This approach differs from clonal depth by explicitly modeling the expected background rate of public TCRs as a function of sequencing depth. We compared the ESLG model score to clonal breadth in the training set and found that the ESLG model score reduced correlation with sequencing depth (total productive templates) compared to clonal depth (Fig. S10). The ESLG model was fit once using UH control repertoires and then frozen before threshold selection and holdout evaluation. The final positive/negative call threshold was set to a specificity of 95% on an independent set of 173 controls only used for threshold selection.

### Definition of HLA variables

All threshold-selection and holdout cohorts had serotyped HLA data. Serotyped HLA was not available for the UH discovery cohort; in that cohort, HLA was inferred directly from the TCR repertoire using a previously published method with high reported accuracy^34^. HLA inference was used only for descriptive analyses of the UH discovery cohort and was not used in discovery of the biomarker score itself. All primary locked evaluations and downstream subgroup analyses used cohorts with serotyped HLA.

### Public disease-associated TCR discovery

We employed a multi-stage design to identify disease-associated TCRs from the training set:

1. We separated the training set by flowcell (3 flowcells total) and computed Fisher’s Exact Test (FET) on each flowcell separately. This reduces potential technical artifacts.
2. We keep only TCRs that have FET p-values less than p1 in at least two flowcells
3. We keep only TCRs that have an FET p-value less than p2 in at least one flowcell
4. This gives us a core set of enriched TCRs
5. The strict p-value threshold ensures these TCRs are relatively common. To pull in less common but still disease-associated TCRs, we employed a clustering strategy. We compute 1-hamming distance clusters (matching V and J genes) around each core TCR and keep TCRs that are within this 1-hamming distance cluster and have an FET p-value less than p3 (FET is computed on the full training set).
6. We performed a grid search over p1=[0.05,0.02,0.01,0.001], p2=[0.01,0.005,0.001,0.0001], and p3=[0.1,0.05,0.01] with 5-fold CV to select the parameters based on the highest average sensitivity at 95% specificity in the held-out folds. A minimum frequency threshold of 2 was enforced for all tests. The selected parameters were: p1=0.01, p2=0.001, p3=0.05.

### Comparator models

Table S3 contains the literature-derived T1D-associated TCRs that were used for comparison to our ADPT TCR set. TCRs were sourced from McPAS and other curated lists^23,24^. Clonal depth was computed for each of these sets at the bioIdentity level (CDR3+Vgene+Jgene). If a TCR was described as binding multiple T1D antigens (e.g. PPI and HIP), the TCR was included in both sets. Our dataset of published TCRs included 895 published GAD-associated receptors, 192 published PPI-associated receptors, 16 published IGRP-associated receptors, 18 published HIP-associated receptors, and 427 other published T1D-associated receptors.

### Multivariable adjustment

The multivariable forest plot in Figure 1 was produced with a logistic regression implemented in statsmodels.Logit. The dependent variable was case status (label). Independent variables were T1D model score, inferred DQ8, inferred DQ2.5, female sex, and age. The continuous variables (score and age) were standardized to mean 0 and standard deviation 1 before model fitting. Odds ratios and confidence intervals were obtained by exponentiating the log-odds coefficients and their Wald confidence intervals.

### Public cluster analysis

Table S2 lists the 264 TCRs comprising the ADPT TCR set. TCRs were grouped into clusters using a graph-based approach in which nodes represent unique CDR3β amino acid sequences and edges connect sequences separated by a Hamming distance of 1, requiring identical TRBJ gene usage while allowing different TRBV genes. Clusters were defined as connected components in this graph, such that all sequences within a cluster are linked through one or more intermediate sequences. This procedure yielded 29 distinct clusters.

Sequence-logo plots were generated for each cluster using the Python package *logomaker*. Cluster-level prevalence in cases and controls, as well as recurrence across cohorts, were computed from a cluster count matrix indicating the presence or absence of each cluster in each sample.

HLA associations were evaluated within T1D cases in the holdout cohort. For each cluster and each serotyped HLA allele or haplotype, enrichment was tested using two-sided Fisher’s exact tests comparing the number of individuals with detectable cluster members in HLA-positive versus HLA-negative individuals. Effect sizes were quantified as odds ratios (ORs). Resulting *P* values were adjusted for multiple testing across all cluster–HLA comparisons using the Benjamini–Hochberg procedure to control the false discovery rate, yielding *q* values. Each cluster was assigned to the HLA allele or haplotype with the lowest *q* value, provided the association met a significance threshold (*q* < 0.05) and had OR > 1.

### DAISY longitudinal timing analysis

The DAISY cohort consisted of 27 participants who eventually converted to T1D (cases) and 25 participants who did not (controls). The controls were also selected as those who did not seroconvert (all autoantibodies negative across all measured timepoints). According to DAISY protocols, participants underwent islet autoantibody testing at 9, 15, and 24 months of age, and annually thereafter; children who became autoantibody positive were then followed more frequently, typically every 3–6 months^7^. We performed TCR sequencing on 4 timepoints from the 27 T1D participants and 25 control participants, totaling 108 TCR repertoires from T1D participants and 100 from control participants. Each participant contributed TCR sequencing data from 4 sampled visits, while islet autoantibodies were measured more frequently over follow-up, with a median of 12 autoantibody-assessment visits per participant.

First autoantibody positivity was defined as the first timepoint where an autoantibody crosses the positivity threshold, for at least one of the four tested autoantibodies (GADA, IA-2A, IAA, and ZnT8A). T-cell positivity was defined as the first sampled timepoint with available TCR sequencing data at which the locked biomarker score exceeded the prespecified locked threshold. Ties between T-cell positivity and serologic milestones were retained as zero lead time.

The lead-time plot defines the per-subject quantity shown on the y axis as (years from first T-cell positivity to diagnosis) - (years from first serologic milestone to diagnosis), such that positive values indicate earlier T-cell detection.

Paired years-before-diagnosis comparisons were plotted for GADA+, IA-2A+, IAA+, ZnT8A+, 2+ autoantibodies, and 1+ autoantibodies versus T-cell positivity. The helper function used a Wilcoxon signed-rank test with alternative = “greater”, reflecting the directional hypothesis that T-cell positivity occurred earlier than the comparator milestone. Smoothed positivity curves were generated with LOWESS (frac = 0.7) over years from diagnosis.

### TrialNet stratification analyses

In all TrialNet stratification analyses, the subgroup threshold was prespecified as the locked biomarker threshold derived independently in the UF threshold-selection cohort; no TrialNet-specific threshold optimization was performed.

For TN09, subject-level 24-month C-peptide area under the curve (AUC) outcomes were derived from trial visit files. Baseline and 24-month AUCs were log-transformed as log(AUC + 1), and treatment– biomarker interaction was assessed using a linear model of the form y_log_ ~ treated × high + log(baseline AUC + 1) + age + sex, where “high” indicates baseline biomarker score above the prespecified threshold.

For TN18, subject-level event time was defined as the earliest of progression to clinical T1D or the second abnormal oral glucose tolerance test (OGTT). Data were expanded into 6-month person–period intervals and analyzed using a complementary log–log generalized linear model with clustered variance by subject:

$${\text{pp}}_{\text{event}}\sim \text{C}\left(\text{interval\_index}\right)+\text{treated}\times \text{high}+\text{age}+\text{sex}.$$

For cross-trial analyses, trial-specific interaction effects were converted to *Z* scores and combined using a weighted Stouffer method, with weights proportional to the square root of the effective sample size for each trial.

For TN18, statistical significance of the treatment–biomarker interaction was additionally assessed using a permutation test. Treatment labels were randomly permuted within biomarker-defined subgroups (high and low) to preserve subgroup structure. For each permutation, restricted mean survival time (RMST) treatment effects were computed within each subgroup up to a prespecified truncation time τ, and the test statistic was defined as the difference in treatment effect between low and high subgroups. Two-sided *P* values were calculated as (|T_perm_| ≥ |T_obs_| + 1)/(N_perm_ + 1).

### Flow-sorted TCR analysis

Flow-sorted tissue samples were sequenced on the immunoSEQ assay. Tissues included pancreatic lymph node (pLN), spleen, inguinal lymph node (iLN), and PBMC samples. In lymphoid tissues, analyses were shown for conventional T cells (Tcon) and regulatory T cells (Treg); in PBMCs, analyses were shown for central memory (CM), naive, Treg, and Tscm subsets. The flow sorting methods for nPOD tissues and bulk PBMCs are described in Rawat et al. and Gomez-Tourino et al. respectively^30,36^.

### Pairing T1D-associated TCRs for deorphanization

PBMCs from T1D patients were stained with antibodies against CD3, CD4, CD8, CD45RA, and CCR7, together with CD14 and CD19 for dump gating and a viability dye. Memory CD4^+^ T cells were isolated by fluorescence-activated cell sorting (FACS) using an L-shaped CCR7/CD45RA gate that excluded naïve CD4^+^ T cells (CCR7^+^CD45RA^+^) while retaining central memory (CCR7^+^CD45RA^−^), effector memory (CCR7^−^CD45RA^−^), and TEMRA (CCR7^−^CD45RA^+^) populations.

Sorted cells were resuspended in RNAlater (Invitrogen) and processed for paired TCRαβ sequencing using the pairSEQ platform, as previously described^37^. Paired full-length TCRα and TCRβ sequences recovered from sorted T cells were used to define relevant receptor pairs for downstream deorphanization workflows.

TCR expression constructs were synthesized and cloned into lentiviral expression vectors by GenScript. Lentiviral particles encoding individual TCRs were used to transduce Jurkat T-cells, and stable TCR-expressing Jurkat T-cells were generated by selection based on surface TCR expression. TCR expression and signaling competence were confirmed prior to downstream assays. Where relevant, TCRs were evaluated in the context of defined HLA class II restriction using antigen-presenting cells expressing the corresponding HLA allele.

### Nucleic acid library-based deorphanization

#### Library design

The two nucleic acid libraries in this study were generated containing T1D-relevant epitopes and all human protein coding genes (HPL), respectively. In each case, individual nucleotide fragments were generated by tiling over coding sequences, where each tile was 150 base pairs long and had 50% overlap with the prior fragment. Fragments with redundant amino acid sequences were discarded, and fragments less than 150bp were padded with a flexible GS linker sequence (GGGGS). For the T1D library, the initial input sequences came from two sources^13,38^ and a collection of additional epitopes from relevant human genes with predicted binding to class II HLA risk alleles. For the HPL, coding sequences (CDS) were downloaded for version GRCh38.p14 of the human reference genome. Using DNAchisel, both libraries were optimized to have GC content between 0.25 – 0.65 and no homopolymers > 5bp. Each library was synthesized by Twist Biosciences, who performed additional codon optimization for expression in human cells.

### Cell engineering and lentiviral delivery

Library inserts were subcloned into a lentiviral expression backbone (pMCSV) designed for intracellular expression and processing of encoded antigen fragments. Inserts were positioned downstream of a CD74-derived signal peptide to promote endosomal trafficking and MHC class II presentation. The vector additionally encoded truncated nerve growth factor receptor (tNGFR) downstream of an internal ribosome entry site (IRES) to enable identification of transduced cells.

K562-derived antigen-presenting reporter cells (APrCs) were engineered to present library-encoded antigens and report T-cell activation. APrCs were transduced to stably express HLA class II molecules (HLA-DQ8, HLA-DQ2.5, or HLA-DRB4) and enriched by fluorescence-activated cell sorting based on surface expression^39^. To enable detection of T-cell activation, APrCs were further engineered with an IL-2 capture system, in which IL-2 secreted by TCR-expressing effector T cells following cognate TCR–pMHC engagement was captured on the APrC surface via an IL-2–specific capture antibody, enabling downstream staining and enrichment, as previously described^37^.

K562 cells were first lentivirally transduced to express an HLA and an IL-2 capture antibody. Next, they were transduced with the nucleic acid library. For each transduction, lentiviral particles were produced by transient transfection of 293FT cells using Lipofectamine 2000 (Thermo Fisher Scientific) and ViraPower packaging mix. Viral supernatant was collected 48 h post-transfection, clarified, and concentrated by polyethylene glycol precipitation (PEG-it, System Biosciences). APrCs were transduced in the presence of polybrene (5 μg ml^−1^) and subjected to spinoculation (2,500 rpm, 32 °C, 1 h). For the T1D library expressed in HLA-DQ8 APrCs, transductions were performed at MOI 2.5 with ~10,000× representation. For the HPL expressed in HLA-DQ8 APrCs, transductions were performed at MOI 30 with ~1,500× representation. The higher MOI for the HPL was selected to maintain sensitivity in the context of substantially greater library complexity. Transduced APrCs were enriched based on tNGFR expression by fluorescence-activated cell sorting and maintained under standard culture conditions.

### T cell co-culture and reporter assay

TCR-expressing Jurkat T cells were co-cultured with APrCs presenting antigen libraries to assess antigen-specific activation. For each TCR, four biological replicates were performed. Cells were co-incubated at an effector-to-target ratio of 1:4 at ~4 × 10^6^ cells ml^−1^ for 5–16 h.

Following co-culture, reporter activation was assessed by detection of IL-2 captured on the surface of APrCs using a PE-conjugated anti–IL-2 antibody (BD Biosciences). IL-2^+^ APrCs were pre-enriched by magnetic separation using anti-PE microbeads (MultiMACS, Miltenyi Biotec) targeting PE-labelled APrCs, and subsequently sorted on a BD FACSAria Fusion cell sorter. A gating strategy was used to select live, singlet APrCs and exclude effector T cells, followed by identification of IL-2^+^ APrCs. Sorted cells were collected for downstream analysis. Antibodies used in FACS are listed in Table S6.

Control experiments were performed to assess HLA restriction and TCRαβ pairing specificity. For HLA-restriction controls, the T1D library was queried with T1D-associated TCRs in HLA-matched and mismatched APrCs. For TCR-pairing controls, TCRβ chains were tested with and without non-cognate TCRα chains. Neither control condition produced antigen enrichment, whereas matched HLA and cognate TCRαβ pairing resulted in HIP enrichment (Supplementary Fig. 11).

### Sequencing and data analysis

Genomic DNA was extracted from sorted IL-2^+^ APrCs and matched unsorted input samples using the QIAamp DNA Kit (Qiagen). Library inserts were amplified in two rounds of PCR. In the first round, insert-specific primers were used to amplify antigen sequences with Q5 polymerase (New England Biolabs). In the second round, Illumina paired-end adapter sequences and an eight-base-pair sample-specific index were incorporated using Multiplex PCR Plus reagents (Qiagen) to enable sample demultiplexing.

Final PCR products were pooled by sample type and purified using SPRIselect magnetic beads (Beckman Coulter). Library quality was assessed using a TapeStation system (Agilent), and concentrations were quantified by qPCR using the KAPA Library Quantification kit (Roche) prior to sequencing. Libraries were sequenced on a NextSeq 2000 system (Illumina).

Raw sequencing reads were processed using Cutadapt (v4.6) to remove vector-derived sequences, retaining only cloned antigen fragments. Trimmed reads were aligned to the reference library using bwa-mem (v0.7.12). Read counts per fragment were obtained using samtools (v1.19), considering only primary alignments with mapping quality ≥20.

Fragments with fewer than 50 reads in control samples were excluded. Read counts were normalized to reads per million (RPM) by adding a pseudocount of 1 to each fragment, dividing by the total read count per sample, and multiplying by 10^6^. Enrichment was calculated as the ratio of RPM in sorted versus control samples and aggregated across replicates (n = 4 per TCR) using the geometric mean. Statistical significance was assessed using a permutation-based maxT procedure with 100,000 permutations to generate a null distribution, and family-wise error rate–adjusted *P* values were calculated. All analyses were performed using custom scripts in Python (v3.10.6).

### T-cell activation assay

TCR sequences were encoded in pcDNA vectors as a single open reading frame (ORF), in the form of the full TCRB sequence followed by an RAKR motif and porcine teschovirus 2A cleavage peptide with the full TCRA sequence after in frame. Capped, polyadenylated mRNA was generated by in vitro transcription from a pcDNA template containing a T7 promoter followed by an AGG transcriptional start sequence. RNA was synthesized using the T7 FlashScribe Transcription Kit (CellScript) with co-transcriptional incorporation of CleanCap AG cap analogs, followed by post-transcriptional poly(A) tailing using the A-Plus Poly(A) Polymerase Tailing Kit (CellScript).

TCRα−/− TCRβ−/− Jurkat cells stably expressing CD4 were transfected with 10 mg *in vitro* transcribed RNA of TCR(s)-of-interest using a Lonza 4D-Nucleofector, SE Cell Line 4D-Nucleofector Kit, program CL-120 (Lonza). K562 cells stably expressing HLA-DQA1*03:01+DQB1*03:02(DQ8), HLA-DQA1*05:01 (DQ2.5) or HLA-DRB4*01:03 were used as antigen-presenting cells. Peptides (>75% purity, Genscript) were dissolved in DMSO, diluted to desired concentration in cell culture media as indicated in figures and pulsed onto K562 cells. TCR-transfected Jurkat cells and peptide-pulsed K562 cells were co-cultured at an effector-to-target ratio of 1:4 overnight and CD54^–^CD4^+^ TCR-transfected Jurkat cells were assessed for CD69 upregulation by flow cytometry. For recombinant protein T-cell activation assays, human proinsulin protein (R&D Systems) was heat denatured at 95°C for 20min and pulsed onto HLA-expressing K562s. TCR-expressing Jurkat cells and protein-pulsed K562s were co-cultured at an effector-to-target ratio of 1:4 overnight and CD54^–^CD4^+^ T cells were assessed for CD69 upregulation by flow cytometry. Antibodies used in flow cytometry are listed in Table S6.

### Positional scanning synthetic combinatorial library (PS-SCL)

A positional scanning synthetic combinatorial library (PS-SCL) was designed for the 13mer base peptide, GG{EA}xx{GLF}xPxxEGG, where x represents an equal mixture of all 20 amino acids. A combinatorial peptide library was created by introducing each naturally occurring amino acid, one at a time at each position from position 3 to 11. Ex. GGAxx{GLF}xPxxEGG, GGCxx{GLF}xPxxEGG, GGDxx{GLF}xPxxEGG, GGExx{GLF}xPxxEGG, … GG{EA}xx{GLF}xPxxVGG, GG{EA}xx{GLF}xPxxWGG, GG{EA}xx{GLF}xPxxYGG.

A 9×20 combinatorial library yields 180 wells of peptides. Negative controls include 25 wells with no peptide. Test peptides and negative controls are tested using three 96-well plates. For quality control, we distribute negative controls evenly within each 96-well plate. To ensure there is no well-effect, we first estimate the correlation between the negative controls and the order in which data is read off the Novocyte. Significant (p-value < 0.05) correlation resulted in the correction of a given plate’s data using the slope coefficient. To correct for potential variation in CD69 activity measurements between plates, we next normalized mean fluorescence intensity (MFI) values within each plate using the following equation:

$$x^{\prime }=\left(x-s_{neg}\right)/m_{neg}$$

where x is the corrected MFI value, *m*_*neg*_ is the average corrected MFI of the negative controls, and *s*_*neg*_ is the standard deviation of the negative controls. Following normalization, we combine data from all plates for subsequent analysis.

We create a position probability matrix (PPM) from corrected MFI values, with a single value per amino acid/position. Using this PPM, we then assign a score to every possible 9-mer in proteomes of interest. Scores are simply the product of each amino acid position’s weighted score from the PPM. For this step, we queried a HIP epitope database, and 9-mer matches with the highest probabilities were prioritized for additional follow-up.

### Spliced peptides database

We created a database of potential *cis*-spliced peptides derived from proinsulin, similar to methods previously reported^8^. All possible 12mer sequences were tiled across proinsulin and joined together in both orientations. We retained all possible spliced peptides for scoring with our PS-SCL results. In addition to the putative *cis*-splicing events, we queried all putative HIPs defined in the nucleic acid deorphanization library design as described above with our experimentally derived PPMs generated from PS-SCL.

### Quantification and statistical analysis

Unless otherwise indicated, statistical testing in the notebook was two-sided. Cohort summary comparisons used Welch t-tests for age, Fisher exact tests for binary HLA variables, and Mann-Whitney U tests for productive templates and clonality. Sensitivity confidence intervals were Wilson intervals. The DAISY paired-timing helper function used one-sided Wilcoxon signed-rank testing (àlternative = “greater”`) for the directional question of whether T-cell positivity preceded each serologic milestone. Timing-correlation panels used Spearman correlation. Trial-specific survival panels in the supplementary figure display Cox-model and log-rank summaries, whereas the principal TN18 interaction panel reports an RMST-based summary and permutation p-value.

## Supplementary Files

This is a list of supplementary files associated with this preprint. Click to download.

- SupplementaryFigures.pdf
